# Changes in physiological arousal during an arithmetic task: profiles of elementary school students and their associations with mindset, task performance and math grade

**DOI:** 10.1038/s41598-024-51683-7

**Published:** 2024-01-18

**Authors:** Ita Puusepp, Tuisku Tammi, Tanja Linnavalli, Minna Huotilainen, Sonja Laine, Elina Kuusisto, Kirsi Tirri

**Affiliations:** 1 Faculty of Educational Sciences, University of Helsinki, Siltavuorenpenger 5 A, Helsinki, Finland; 2 Cognitive Science, Department of Digital Humanities, University of Helsinki, Helsinki, Finland; 3 Cognitive Brain Research Unit, Faculty of Medicine, University of Helsinki, Helsinki, Finland; 4 Centre of Excellence in Music, Mind, Body and Brain, University of Helsinki, Helsinki, Finland; 5 Faculty of Education and Culture, Tampere University, Tampere, Finland

**Keywords:** Psychology, Human behaviour

## Abstract

Task-related change in physiological arousal is suggested to reflect active involvement with the task. While studies often examine such task-related changes in arousal as averaged across the entire task, the present study focused on temporal changes in arousal during a task. More specifically, we investigated changes in elementary school students’ physiological arousal during an arithmetic task and associations between these changes and students’ mindset, performance on the task, and math grades. We used a person-oriented approach to analyze the tonic electrodermal activity of 86 fourth graders, recorded while they were working on an arithmetic task. With model-based clustering of students’ on-task electrodermal activity, we identified three groups of students with differing temporal dynamics of physiological arousal during the task: *Increasing Arousal*, *Decreasing Arousal* and *Decreasing and Increasing Arousal*. The *Decreasing Arousal* profile contained more students classified as holding a *Fixed Mindset Tendency* than would be expected if physiological profile membership and mindset tendency were independent. The *Increasing Arousal* profile performed better on the task than the *Decreasing Arousal* profile. No association was found with math grades. These results provide a new insight into individual differences in temporal patterns of on-task physiological arousal.

## Introduction

Mastering complex and cognitively demanding topics requires students to not give up when they experience difficulties, but to sustain active engagement with the learning material also when it feels effortful^1^. While the use of observational measures has contributed to our understanding of visibly observable differences in students’ learning behavior e.g.,^2^, less is known about *covert* differences in students’ active engagement during educational activities—differences in their motivational processes, when no visually observable differences in learning behavior are present. Furthermore, students’ engagement with a task is a dynamic process—emotions, levels of interest and expenditure of effort are not necessarily the same at the beginning of the task as they are towards the end or even in the middle of the task. The dynamics of students’ self-reported on-task motivational indicators have been shown to be associated with their general motivational tendencies^3^ and task performance^4^. Nonetheless, self-reports are subject to social desirability e.g.,^5^, are often used retrospectively, or require interrupting participants’ during the task. Continuously recorded physiological data, though, has the potential to shed light on both the covert as well as dynamic aspects of individuals’ motivational processes as they unfold during specific activities^6,7^. Namely, one’s motivational and affective processes are reflected in the activity of one’s autonomic nervous system^7–10^.

The sympathetic axis of the autonomic nervous system is responsible for preparing the body for action^11^. Therefore, greater sympathetic activity—from here on referred to as greater physiological arousal—reflects more intense energization of behavior or motivational activation. Such activation entails both avoidance as well as approach motivation^10,12^. While avoidance motivation is the energization of behavior by, or away from, negatively evaluated stimuli (e.g., an urge to distance oneself from a threatening situation), approach motivation entails the energization of behavior by, or toward, positively evaluated stimuli (e.g., an urge to engage in an enjoyable activity)^13^. As such, indicators of physiological arousal have been used in both studies focusing on constructs related to avoidance as well as to approach motivation^9,14–16^. Researchers examining physiological arousal in the context of performing mental tasks have observed participants’ arousal to change notably from resting baseline to the active task situation. As such a task-related change in arousal—task-related-activation—has been linked to performance on the task (the greater the task-related change in arousal, the better the performance), it has been assumed to reflect active involvement with the task^8,17–20^. Consistently with this, higher physiological arousal has been shown to associate with greater self-reported mental effort during tasks^21^.

The majority of studies examining on-task autonomic nervous system activity have mainly focused on the mean levels of such activity across the entire task, without investigating on-task temporal changes. This might be due to the relatively short duration of the experimental tasks used in such studies (often lasting 3–5 min^8,17–19^). Nonetheless, there are some studies that have investigated temporal changes in on-task autonomic nervous system activity and these have shown such fluctuations to be related to individual’s overall motivation^22,23^.

Furthermore, differences in physiological arousal during different task phases^24^ and variability in its fluctuation during multiple task blocks have been reported^18^. Such individual variability and differences in participants’ on-task arousal have been suggested to reflect differences in participants’ on-task effortful engagement and its fluctuations^18,22,23^. Studies utilizing self-reports have, indeed, indicated that students’ motivational indicators and emotions fluctuate during tasks^25,26^. Furthermore, significant individual variability in these fluctuations has been shown^4,25^, with one study identifying subgroups of students displaying differing on-task motivational trajectories^27^. Moreover, Tapola and colleagues^3^ demonstrated that students’ situational interest during a learning task either decreased or increased depending on their general motivational tendencies. Therefore, it is probable that students differ not only in their mean levels of task-related activation, but also in how their arousal changes while they work on the task, with these fluctuations being reflective of on-task changes in their motivational activation. In the present study, on-task changes in students’ electrodermal activity (EDA)—a sensitive indicator of sympathetic activity^28^—are used to indicate changes in students’ task-related activation^8,17,18,21^. EDA entails changes in the electrical properties (conductance and resistance) of the skin that result from sweat secretion by eccrine sweat glands^28^. EDA is often divided into a slower fluctuating tonic component—skin conductance level (SCL)—and a more rapidly fluctuating phasic component—skin conductance response (SCR). While the latter is often used to study responses to brief, discrete stimuli, SCL is used to investigate general states of arousal and alertness^28^. Additionally, SCL has been suggested to be a useful index when measuring processes related energy mobilization^28^. Furthermore, research among adults as well as children has suggested task-related changes in SCL to reflect active involvement with mental tasks^8,17–20^. Therefore, in the present study we focus on changes in students’ SCL during a task, with higher SCL indicating greater sympathetic activity and, thus, higher levels of arousal, alertness, and activation^28^.

As research has indicated that the dynamics of students’ on-task motivational indicators are associated with students’ general motivational tendencies^3^, we explore how temporal dynamics of students’ arousal are associated with their mindset. Mindsets refer to the beliefs about the malleability of human abilities. These beliefs range from fixed, the belief that human attributes are static, to growth, the belief that these attributes are malleable and can be developed^29^. Mindset is suggested to organize one’s goals, attributions, and views on effort into a meaning system—a framework that shapes one’s thinking and behavior^30–32^. As such, compared to a more growth mindset, a fixed mindset is associated with holding negative *effort beliefs*—believing effort to be futile as a means of enhancing ability rather than appreciating its utility^33^. Additionally, students’ mindset orients their *goal endorsement*: in tradeoff situations, students with a fixed mindset tend to endorse goals to prove their ability (performance goals) rather than goals to obtain mastery (mastery goals)^33^. Mindsets have been shown to associate with aspects of task-related motivational activation as indicated by behavioral measures: a more growth-oriented mindset has been associated with greater mental effort^34^ and sustained engagement during a task^35^. To our knowledge, there are two studies that have focused on autonomic nervous system activity during a task in relation to mindsets, finding no association between the two^24,36^. In these two studies, autonomic nervous system activity averaged across an entire task was inspected, while in the present study the focus is on on-task fluctuations in such activity. Namely, mindset-related differences in sustained effortful engagement with the task could be reflected in differences in on-task changes in arousal.

We also explore how physiological arousal during a task is associated with achievement outcomes: performance on the task and grades. Increases in approach-motivation-related constructs during a task have been shown to predict better task performance^4,25^. Additionally, higher task-related activation as indicated by physiological arousal has been associated with better task performance^8,17–20^. As to grades, they reflect both students’ skills and aspects of their learning behavior and motivation in the respective subject^37^. When examining associations with these outcomes, we controlled for the effects of prior achievement.

The aim of the present study, which is part of the project Copernicus—Changing Mindsets about Learning: Connecting Psychological, Educational and Neuroscientific Evidence, was to explore the temporal dynamics of elementary school students’ physiological arousal as they work on an arithmetic task and the associations between such dynamics and students’ mindset, task performance, and math grade. The study focused on fourth graders’ arousal during a math task as many students consider math to be one of the most important school subjects^38,39^, and after the first years of elementary school the concepts covered in math increase notably in complexity. This requires students to be persistent in their active engagement during the learning process, even when it feels effortful^1^.

According to the studies mentioned above, motivational processes are dynamic and linear trends in on-task physiological arousal cannot be assumed, while notable individual variability in the temporal changes in such arousal and self-reported motivational indicators have been demonstrated. Therefore, we used a person-oriented approach, which enables (1) to examine dynamics of arousal without assuming linear temporal trends and (2) to possibly clarify previous findings indicating notable individual variability in these dynamics. Namely, this approach does not assume homogeneity in terms of the patterns of studied variables^40^. Therefore, it enables to identify groups of students displaying differing temporal dynamics of arousal. As to students’ mindsets, we used a person-oriented approach to classify students based on multiple differently assessed indicators of their mindset meaning system: self-reported mindset and effort beliefs as well as a proximal behavioral indicator of goal endorsement in a tradeoff situation. We adopted this approach as previous research has shown the use of multiple indicators of mindset meaning system among young elementary school students to be useful^41^.

The research questions and hypotheses were as follows:

RQ1. How do students differ in the temporal dynamics of their physiological arousal while working on an arithmetic task?

(H1) We expect to identify multiple groups of students displaying differing temporal dynamics of physiological arousal. Namely, research indicates certain general trends—combined with notable individual variability—in the temporal changes in self-reported on-task motivational indicators and emotions^3,4,25,27^ as well as changes in physiological arousal during a task and individual variability in such changes^18,24^.

RQ2. How do groups of students with different temporal dynamics of physiological arousal during the task differ in

- mindset meaning system,
- achievement outcomes (performance on the arithmetic task and math grade)?

We expect physiological arousal profile membership to be associated with mindset (H2)^3^ and achievement outcomes (H3)^4,25,27^.

## Methods

### Participants

A total of 104 participants from two Finnish public elementary schools in the Helsinki metropolitan area were recruited for the longitudinal Copernicus research project. However, due to withdrawal from the longitudinal project, the participants of the present study were 102 fourth graders (49 identified as girls, 47 as boys, and 6 responded “Other” or did not report their gender; *M*_age_ = 10 years, *SD* = 0.4, range = 9–12). Of these, 97 students' complete questionnaire data and physiological data from 86 students were obtained (44 identified as girls, 39 as boys, and 3 responded “Other” or did not report their gender; *M*_age_ = 10 years, *SD* = 0.5, range = 9–12 years). This resulted in 82 students with both questionnaire and physiological data. The criterion for analysis of the 86 students’ physiological data was information on at least 50% of their EDA in the arithmetic task. The reasons for missing EDA data were a noisy signal and a technical issue during the data recording. In turn, the reasons for missing questionnaire data were absence from school on the days of questionnaire administration and uncompleted surveys.

### Procedure

Students’ participation in the study was voluntary, and written parental consent was obtained. Students and their parents were informed about the research procedures and their right to withdraw at any time during the study. The study was approved by the University of Helsinki Ethical Review Board and the study was carried out in accordance with the guidelines and regulations of the Ethical Review Board and the Declaration of Helsinki. The students were compensated for their participation in the Copernicus research project with sweets and stickers. The participants completed an electronic questionnaire containing the mindset and effort beliefs scales and indicated their goal endorsement during a regular school lesson in the autumn semester of their fourth grade. This took approximately 40 min. The recordings of EDA and electroencephalography (not reported in this study) were performed by a researcher in a separate room on the school premises during regular school hours (the room temperature was set to 21 °C, with possible fluctuations between 18 and 22 °C). Before the recordings, the students were briefed about the experiment and reminded of their right to withdraw their participation at any moment. The participants were seated during the recording, with their non-dominant hand with attached electrodes placed on the table in front of them. After placement of electrodes, the participants were given detailed instructions concerning the experimental task, followed by a practice block of the task. The participants were additionally instructed not to speak during the experiment and to keep their non-dominant hand still and relaxed. The entire procedure lasted 60–75 min per participant, with the time between the electrode placement and start of the recording being approximately 6–8 min, and the physiological recording lasting approximately 20 min.

### Materials

#### Arithmetic task

**Figure 1:**
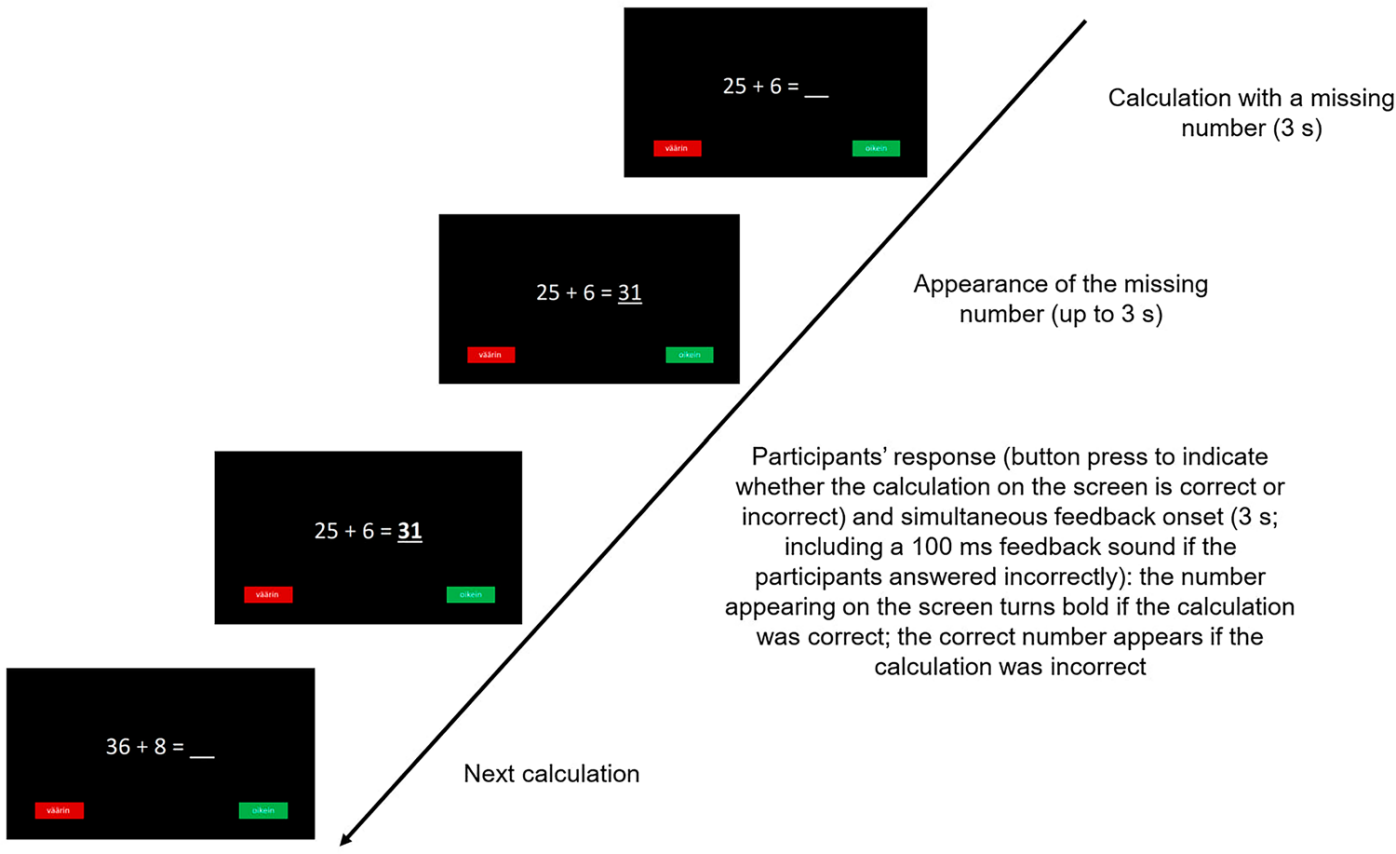
Sequence of events in a trial of the arithmetic task*.* A modified version of the previously published figure in Puusepp and colleagues^42^.

Participants’ EDA was recorded during the completion of a two-alternative choice arithmetic task consisting of 93 trials (Fig. 1). Each trial consisted of an arithmetic calculation with one number missing. The calculation was presented on the computer screen for 3 s, after which either a correct or incorrect answer was presented in the place of the missing number for a maximum of 3 s. During this 3 s response window, the participants used their dominant hand to press one of two buttons on the response box to indicate whether they thought the number that had appeared in the calculation was the correct or incorrect answer. If the number on the screen was an incorrect answer, after the participant’s response this incorrect answer changed into the correct answer, which was displayed on the screen for 3 s. If the number on the screen was the correct answer, this answer was shown in bold on the monitor for 3 s right after the participant’s response. To ensure that the participants were aware they had made a mistake, their incorrect responses were immediately followed by a feedback tone lasting 100 ms. If the participant failed to press a button during the 3 s response window, a timeout message appeared on the monitor for 3 s before the calculation of the next trial appeared. Before the actual task and EDA recording, the participants completed a practice block of 10 trials. Based on their performance during the practice block, the participants were administered either an easier (0–5 trials answered correctly) or a more difficult version (6–10 trials answered correctly) of the task to ensure that the task was sufficiently, but not overly, challenging for the participants. The actual task consisted of two blocks (46 and 47 trials, respectively) with a total of 93 trials. The 93 trials (48 correct calculations and 45 incorrect calculations) were presented in random order to each participant to randomize intra-individual fluctuations in perceived on-task difficulty. Between the two blocks, the children were permitted a short refreshment pause. The calculations in the arithmetic task comprised addition, subtraction, multiplication, and division. After completing the task, the participants indicated their perception of the difficulty of the task on a single item on a 5-point scale (1—*very difficult*…5—*very easy*).

#### Electrodermal activity

EDA was acquired using LiveAmp (Brain Products GmbH, Germany) at a 250 Hz sampling rate using two Ag–AgCl electrodes. The electrodes were attached to the palmar surfaces on medial phalanges of the second and fourth finger of the non-dominant hand using TD-246 paste (0.5% saline in a neutral lotion style base; Discount Disposables, USA) and adhesive skin tape. Two sets of reusable electrodes were used to measure EDA of all of the participants, with the electrodes being cleaned in between the measurements.

#### Mindset meaning system

To assess students’ *math ability mindset*, the four Entity Theory statements from the Implicit Theories of Intelligence Scale^29^ were adapted to be math-specific (e.g., “You have a certain amount of math intelligence, and you cannot really do much to change it.”). *Effort beliefs* were measured using five negatively phrased statements (e.g., “If you have to work hard on some problems, you’re probably not very good at them.”) from the Effort Beliefs Scale^33^. For both the mindset and effort beliefs instruments, participants indicated how much they agreed with each statement by marking one of six circles that varied in size, corresponding to a range of agreement from not at all to really a lot, which then mapped to a 6-point scale. Confirmatory factor analysis with two correlated factors (mindset and effort beliefs) showed good fit: χ^2^(26) = 33.26 (*p* = 0.155); CFI = 0.98, TLI = 0.97, RMSEA = 0.054, SRMR = 0.049. Two average scores were used, with higher scores indicating a greater growth mindset and more positive effort beliefs. The internal reliabilities of these instruments were good (Cronbach’s α for mindset was 0.86, while for effort beliefs it was 0.73). For *goal endorsement in math in a tradeoff situation,* the students were, after working on a difficult math task (see below), asked to indicate their endorsement of one goal over the other: whether they wanted to subsequently work on a more challenging task to learn more or an easier task to definitely succeed. The difficult math task preceding this choice consisted of three demanding arithmetic exercises that were intended to be above the students’ grade level. The exercises were chosen, and their level evaluated, by two elementary school teachers from one of the schools participating in this study. The students were assigned a maximum of 5 min to work on the task, after which the online system automatically moved on to the next page of the online questionnaire, where the students first indicated whether they had perceived the previous task as difficult or easy and subsequently indicated their goal endorsement (see Fig. S1 for the challenging arithmetic task and the following question regarding perceived difficulty as well as goal endorsement).

#### Achievement outcomes

*Task performance* was calculated as the percentage of correctly answered trials. The participants’ *math grades* were provided by the schools upon request. Math grades both from the spring semesters of participants’ 3rd and 4th grades were obtained to enable controlling for previous achievement. In the 3rd grade, both participating schools used a verbal grading system with five levels indicating how well the student had achieved the learning goals of the semester. These levels were recoded into a numerical scale from 1 to 5. In both schools, 4th grade grades were based on a numerical grading system with a scale from 4 to 10. For both grade levels, higher numbers indicate better achievement.

### Overview of data analysis

The EDA data were processed with MATLAB R2019a software (Mathworks, Natick, MA, USA) with the Ledalab toolbox^43^. The signal was downsampled to 10 Hz and decomposed into the tonic and phasic component using Continuous Deconvolution Analysis with two-step parameter optimization (CDA)^43^. The tonic component of EDA (SCL) was then extracted for further analyses, and the data were visually inspected for artefacts.

For RQ 1, participants’ raw SCL was cut into four blocks: SCL during trials (1) 1–22, (2) 23–46, (3) 47–70, (4) 71–93 (the average duration of a block was 181 s, *SD* = 8 s). Subsequently, mean SCLs for each of the four blocks were calculated and standardized within individuals. The presence of outliers was visually inspected, with no extreme outliers found. Subsequently, model-based latent profile analysis (LPA), which enables the detection of homogeneous subgroups based on multiple indicator variables, was used to identify subgroups of students with different temporal dynamics of physiological arousal during the task. The subject-wise standardized mean SCLs of the four blocks were utilized as indicator variables and the *TidyLPA* package^44^ in R (version 4.3.0) was used for the LPA. Of the 86 participants whose physiological data was used for the analysis, six had missing SCL data from block 1, five from block 2, six from block 3, and nine from block 4 due to a noisy signal. We used the *missForest* package^45^ to impute these missing values. We also conducted model-based clustering with a sample consisting of only participants with the complete EDA data. The overall results did not differ from the ones reported here (see Table S1 and Fig. S2). LPA models (equal variances across classes and covariances fixed at zero) with one to five physiological profiles were explored. The best solution was determined by visually inspecting the SCL distributions of the profiles in different models and by considering interpretability, sample sizes of profiles and fit metrics (Akaike Information Criterion [AIC], Bayesian Information Criterion [BIC], sample-size adjusted BIC [SABIC], entropy and values of bootstrap likelihood ratio test [BLRT]).

For RQ2 regarding the associations between physiological profile membership and mindset (H2), students were first classified based on their mindset meaning systems. This classification was performed based on the results of model-based clustering on the three assessed meaning system indicators (mindset, effort beliefs, and goal endorsement in a tradeoff situation). This clustering enabled the identification of groups of students with differing mindset meaning systems. The *clustMD* package^46^, which is suitable for clustering mixed data, was used. Only observations with complete data on the three mindset meaning system indicators were used, with standardized continuous variables. Models (equal variances across classes and covariances fixed at zero) with one to four mindset profiles were explored, and the best solution was determined by considering theoretical interpretability and BIC as well as by visually inspecting the solutions. Subsequently, crosstabulation with Fisher’s exact test (*gmodels* package)^47^ was used to detect associations between mindset and physiological profiles. In the case of a significant result, we used the Bonferroni correction to adjust the adjusted standardized residual cutoff value indicating a significant difference (an absolute value of 1.96 for a single comparison) for the number of the cells in the analysis, as recommended by MacDonald and Gardner^48^. Cramer’s V (*φ*_*c*_) was used to indicate effect size regarding differences in mindset profile membership (0.10, 0.30, and 0.50 interpreted as being indicative of small, medium, and large effects, respectively)^49,50^.

To inspect the physiological profile differences in achievement outcomes (H3), separate linear regressions with task performance and 4th grade math grade as dependent variables and profile membership as a categorical independent variable were performed. When inspecting associations between profile membership and 4th grade math grade, we controlled for participants’ previous math grade. Concerning associations with task performance, we controlled for the assigned difficulty level of the experimental task (based on the participants’ performance on the practice block) and their previous semester math grade. For these analyses, math grades were standardized within schools for both grade levels. In the case of significant effects, pairwise comparisons with the Bonferroni-Holm adjustment for multiple comparisons were performed. Partial *η*^2^ was used to indicate effect size (0.01, 0.06, and 0.14 interpreted as being indicative of small, medium, and large effects, respectively)^49,50^. Alpha level was set at *p* < 0.05. Results of supplementary analyses on associations between physiological profile membership and participants’ gender and the school they attend are reported in Supplementary Information (Figs. S3 and S4).

### Ethics statement

This study involving human participants was reviewed and approved by the University of Helsinki Ethical Review Board. Written informed consent to participate in this study was provided by the participants’ legal guardian/next of kin.

## Results

The descriptive statistics and correlations between the study variables are presented in Table 1. The mean of the raw on-task SCL was 11.40 μS (*SD* = 3.65; range = 3.69–25.98) for block 1, 11.37 μS (*SD* = 3.65; range = 3.46–25.29) for block 2, 11.32 μS (*SD* = 3.62; range = 4.24–24.54) for block 3, and 11.13 μS (*SD* = 3.75; range = 3.75–24.66) for block 4. Based on their performance on a practice block of the arithmetic task, 35 students were assigned an easier version of the task and 63 a more difficult version. There was no significant difference in perceived difficulty of the task between these two groups (*p* = 0.306).

**Table 1:** Pairwise correlations between and descriptives of the study variables. ^†^p < 0.10; *p < 0.05; **p < 0.01; ***p < 0.001. Correlations with goal endorsement and SCLs are Spearman rank-correlations, otherwise Pearson correlations are presented. The SCL of each block is the within-individual standardized averaged SCL across the indicated trials. ^a^The number of students who pursued mastery goal in a tradeoff situation. The column numbers refer to variables with the same number in the rows.

Variable	1	2	3	4	5	6	7	8	9	10
1. SCL_block 1_ (trials 1–22)										
2. SCL_block 2_ (trials 23–46)	0.14									
3. SCL_block 3_ (trials 47–70)	− 0.65***	− 0.39***								
4. SCL_block 4_ (trials 71–93)	− 0.62***	− 0.64***	0.20^†^							
5. Mindset	− 0.02	− 0.35**	0.00	0.30**						
6. Effort beliefs	− 0.07	− 0.30**	0.20^†^	0.36**	0.61***					
7. Endorsement of mastery over performance goal	− 0.21^†^	− 0.38***	0.26*	0.38***	0.32**	0.46***				
8. Task performance (%)	− 0.23*	− 0.38***	0.29**	0.29*	0.27**	0.30**	0.21*			
9. 3rd grade math grade	− 0.31**	− 0.11	0.21†	0.26*	0.24*	0.32**	0.31**	0.42***		
10. 4th grade math grade	− 0.10	− 0.17	0.02	0.21^†^	0.45***	0.25*	0.39***	0.42***	0.73***	
*N*	80	81	80	77	99	99	97	98	101	100
*M* (*SD*)	− 0.114 (0.945)	− 0.238 (0.740)	0.411 (0.690)	− 0.028 (0.944)	4.34 (1.20)	4.23 (0.99)	56^a^	60 (11)	4.17 (0.87)	8.61 (0.95)
*Min*	− 1.489	− 1.487	− 1.316	− 1.497	1.00	1.60	–	39	2	6
*Max*	1.377	1.495	1.419	1.467	6.00	6.00	–	90	5	10

### Physiological profiles

**Figure 2:**
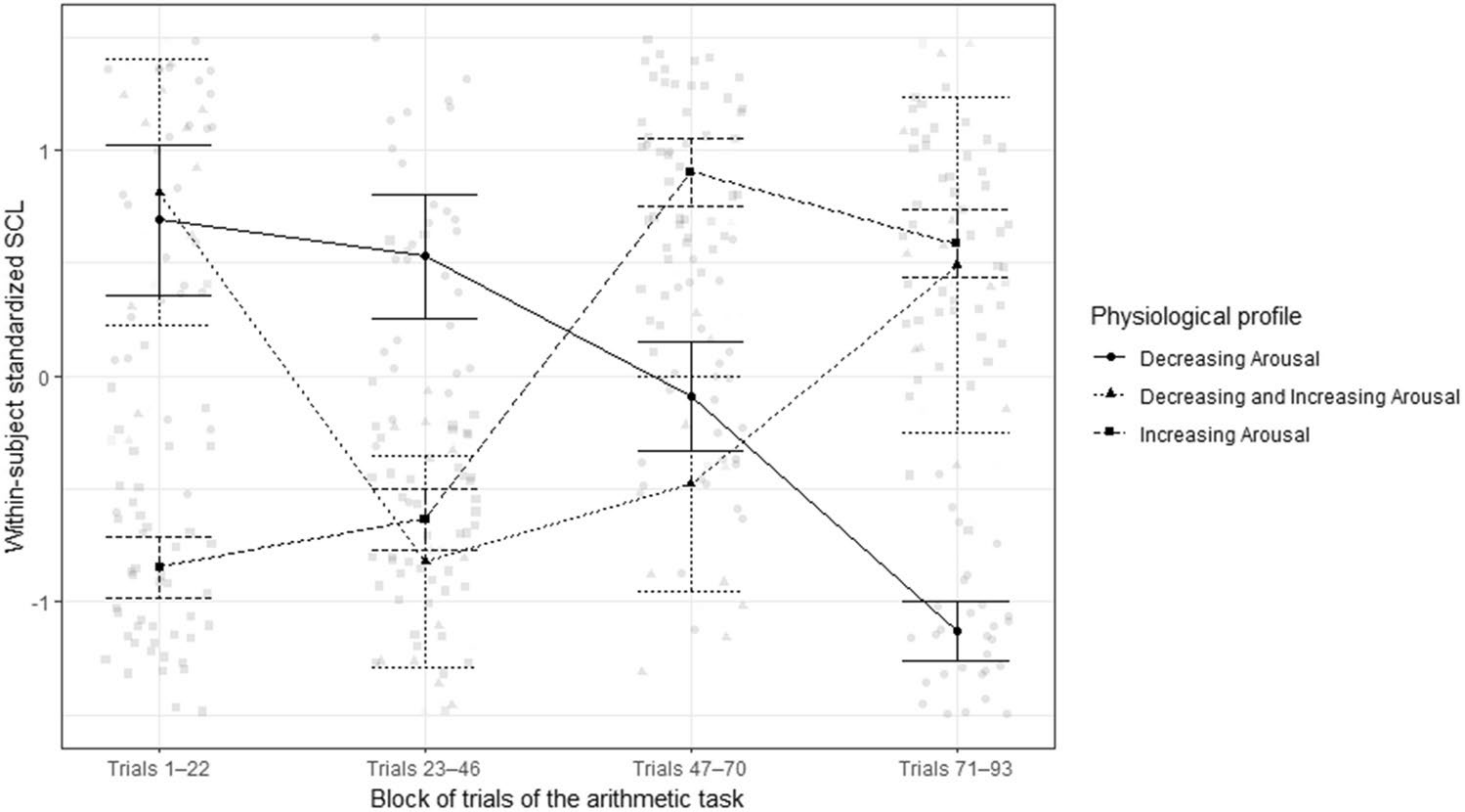
Line graph comparing the three retained physiological profiles regarding SCL (standardized within individuals) during the arithmetic task. Observations are shown as points and 95% confidence intervals of SCL for each group at each trial block as bars.

Latent profile analysis (LPA) with one to six physiological arousal profiles was conducted. Based on the model fit indices, visual inspection of the solutions, and taking into account the sample sizes of the profiles (Table 2), the three-profile model was retained for further analysis. The three-profile solution displayed the highest entropy, and while AIC, SABIC and BIC dropped notably from the two- to the three-profile solution, solutions with more profiles resulted in only slight decreases in AIC, BIC and SABIC, with declining entropy and minimum probability of profile membership. Visual inspection of the SCL distributions of the profiles of different solutions indicated that the three-profile solution contained groups of students with qualitatively different temporal dynamics of SCL (see Fig. S5). The three profiles were named (1) *Decreasing Arousal* (n = 29), (2) *Decreasing and Increasing Arousal* (n = 11), and (3) *Increasing Arousal* (n = 46; Fig. 2, Table 3)*.*

**Table 2:** Fit indices of the LPA solutions of physiological arousal. Selected model in bold.

Number of profiles	Log likelihood	AIC	BIC	SABIC	BLRT *p*-value	Entropy
1	− 419.84	855.69	875.32	850.08	–	1.00
2	− 338.12	702.23	734.14	693.12	0.01	0.96
**3**	**− 303.66**	**643.33**	**687.51**	**630.72**	**0.01**	**0.97**
4	− 289.90	625.81	682.26	609.69	0.01	0.91
5	− 272.58	601.17	669.89	581.55	0.01	0.91

**Table 3:** Descriptive statistics of physiological profiles.

Within-subject standardized SCL	Physiological profile								
	Decreasing arousal (n = 29)			Decreasing and increasing arousal (n = 11)			Increasing arousal (n = 46)		
	M (SD)	95% CI		M (SD)	95% CI		M (SD)	95% CI	
		LL	UL		LL	UL		LL	UL
Block 1 (trials 1–22)	0.69 (0.61)	0.46	0.92	0.79 (0.59)	0.39	1.18	− 0.85 (0.43)	− 0.97	− 0.72
Block 2 (trials 23–46)	0.53 (0.53)	0.33	0.73	− 0.81 (0.52)	− 1.16	− 0.46	− 0.64 (0.44)	− 0.43	− 0.11
Block 3 (trials 47–70)	− 0.09 (0.51)	− 0.29	0.11	− 0.51 (0.56)	− 0.89	− 0.13	0.91 (0.37)	0.80	1.02
Block 4 (trials 71–93)	− 1.13 (0.28)	− 1.23	− 1.03	0.53 (0.61)	0.12	0.94	0.58 (0.46)	0.44	0.71

### Mindset profiles

Solutions with one to four student groups based on multiple mindset meaning system indicators (mindset, effort beliefs, and goal endorsement in a tradeoff situation) were examined. Based on BIC (BIC_1 profile_ = − 708.13, BIC_2 profiles_ = − 683.48, BIC_3 profiles_ = − 692.73, BIC_4 profiles_ = − 710.52), visual inspection of the distributions of indicator variables, and posterior probabilities of group membership and theoretical interpretability, the two-profile solution was used to classify students into different mindset tendencies (see also Figs. S6 and S7). The two profiles were named *Growth Mindset Tendency* and *Fixed Mindset Tendency* (Table 4).

**Table 4:** Descriptive statistics of mindset profiles.

Mindset meaning system indicator	Mindset profile					
	Growth mindset tendency (n = 64)			Fixed mindset tendency (n = 33)		
	M (SD)	95% CI		M (SD)	95% CI	
		LL	UL		LL	UL
Mindset	4.98 (0.80)	4.78	5.18	3.13 (0.90)	2.81	3.44
Effort beliefs	4.74 (0.62)	4.59	4.90	3.24 (0.85)	2.94	3.54
Endorsement of mastery goal over performance goal (n [%])	48 (75%)	–	–	8 (24%)	–	–

### Differences between physiological profiles in mindset tendencies and achievement outcomes

#### Mindset tendencies

**Figure 3:**
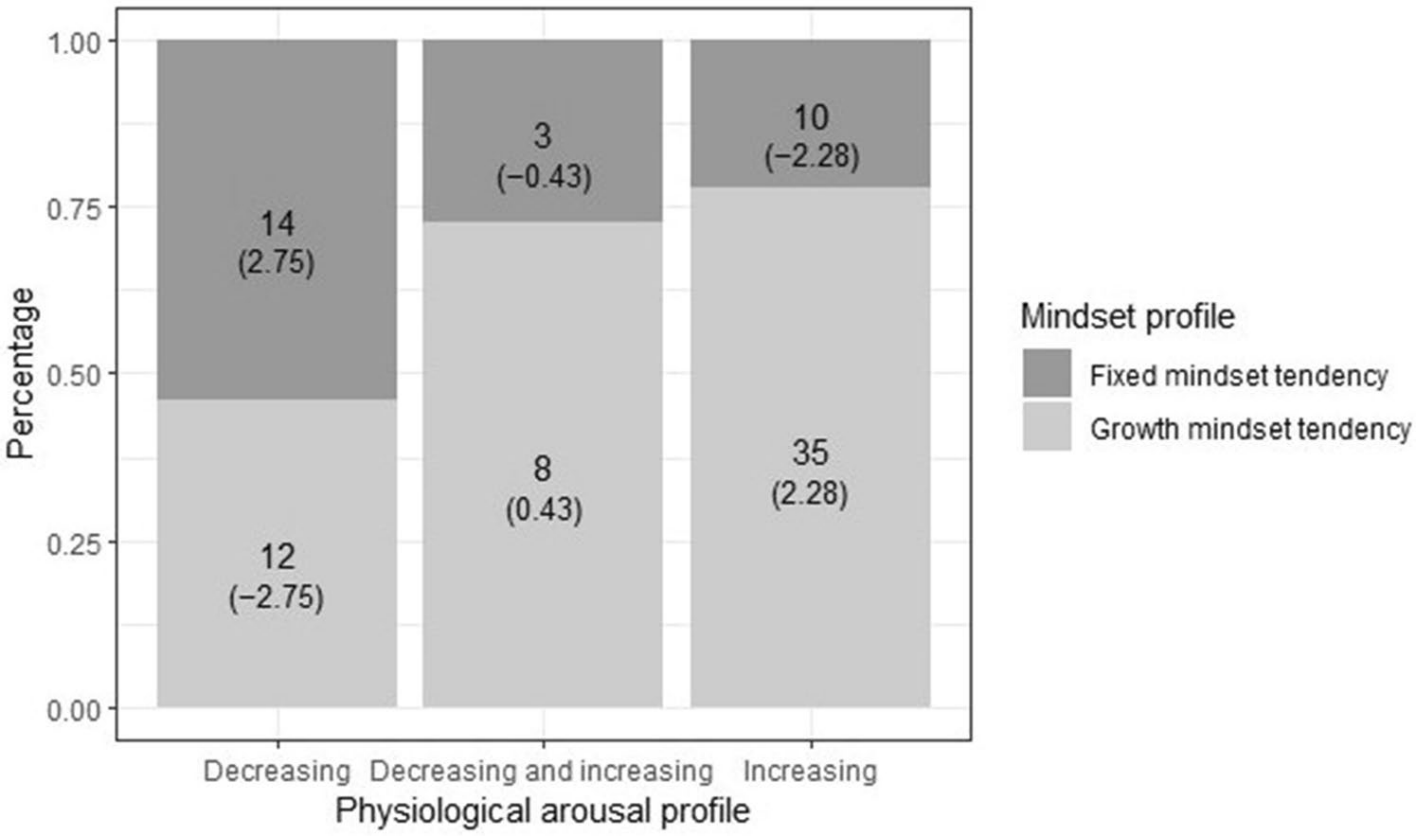
Bar plot representing the distribution of mindset tendencies across physiological profiles. The figure displays observed counts with adjusted standardized residuals indicated in brackets.

Crosstab with Fisher’s exact test indicated that the physiological profiles and mindset tendencies were not independent (*p* = 0.025, *φ*_*c*_ = 0.31, Fig. 3). The adjusted standardized residuals (cutoff indicating statistical significance adjusted to ± 2.64 based on the number of cells) revealed that the *Decreasing Arousal* profile contained significantly more students than expected with a *Fixed Mindset Tendency* and significantly less students than expected with a *Growth Mindset Tendency* (Fig. 3). By contrast, membership of the *Decreasing and Increasing Arousal* profile was not associated with mindset tendency. As to the *Increasing Arousal* profile, while it contained more *Growth Mindset Tendency* students and fewer *Fixed Mindset Tendency* students, this was below the adjusted level of statistical significance (Fig. 3).

#### Achievement outcomes

Crosstab with Fisher’s exact test indicated that the physiological profile membership was not associated with the version of the assigned arithmetic task (*p* = 0.346, *φ*_*c*_ = 0.15). Moreover, based on linear regression with 4th grade math grade as the outcome and physiological profile and previous math grade as predictors, the physiological profiles did not differ significantly in terms of math grade (*F*[2, 80] = 0.96, *p* = 0.388, *η*^2^_*p*_ = 0.02). By contrast, based on linear regression with task performance as the outcome and physiological profile, previous math grade, and version of the task as predictors, the physiological profiles differed in terms of task performance (*F*[2, 80] = 7.21, *p* = 0.001, *η*^2^_*p*_ = 0.15). Post hoc comparisons revealed that the *Increasing Arousal* profile group performed significantly better on the arithmetic task (*M* = 63%, *SE* = 2%) than the *Decreasing Arousal* group (*M* = 55%, *SE* = 2%; *p* = 0.007), while the performance of the *Decreasing and Increasing Arousal* group did not differ from other groups (*M* = 59%, *SE* = 3%; *p*s = 0.481).

## Discussion

The present study explored the temporal dynamics of elementary school students’ physiological arousal during an arithmetic task. Using a person-oriented approach, we identified three groups of students with distinct temporal dynamics of physiological arousal during the task: *Decreasing Arousal*, *Decreasing and Increasing Arousal*, and *Increasing Arousal* (H1). Furthermore, we found that the temporal dynamics of students’ arousal during the task was linked to their mindset and task performance, but not to math grade. Although no previous study has used a person-oriented approach to explore temporal patterns of physiological arousal while engaging with a task, the identification of profiles with differing patterns is in line with research that has shown notable individual variability in changes in EDA^18^ and self-reported motivational constructs during task completion^25–27^.

Concerning mindsets, most students belonged to the *Growth Mindset Tendency* profile, characterized by a growth mindset and positive effort beliefs, and most students in this group endorsed a mastery goal in a tradeoff situation (either a mastery or performance goal). The smaller *Fixed Mindset Tendency* profile was characterized by a more fixed mindset and more negative effort beliefs, although these self-reported indicators did not fall at the extreme fixed mindset and negative effort belief ends of the scales. Students possibly perceived the fixed mindset and negative effort beliefs statements as socially undesirable^51^. Most students in this profile group nevertheless endorsed a performance goal in a tradeoff situation. Overall, these mindset groups—a *Fixed* and a *Growth Mindset Tendency—*are in line with earlier person-oriented studies on mindsets^52–55^, the majority of which have consistently demonstrated the presence of distinct groups of students described by a growth mindset and a fixed mindset. Additionally, notably smaller groups of students with moderate or somewhat contrasting mindset meaning system indicators have been identified (e.g., holding a growth mindset but endorsing performance goals)^52–55^. Nevertheless, in those studies goal endorsement was not assessed in a tradeoff situation. Namely, it is specifically in such situations—where one goal must be chosen over another—that a growth mindset is suggested to be linked to endorsement of mastery goals^32^. In the present study, we used a behavioral assessment of goal endorsement in a tradeoff situation. Nonetheless, as previous studies have shown profiles with mixed mindset indicators to be much smaller than growth and fixed profile groups, the sample in the present study was possibly too small to identify such smaller groups.

As expected, we found that membership of physiological profiles and mindset tendencies was not independent (with a medium-sized effect; H2). The students in the *Decreasing Arousal* profile were more likely characterized by the *Fixed Mindset Tendency* than would be expected by chance. As higher tonic arousal has been shown to associate with greater self-reported mental effort^21^, the decrease in arousal displayed by students in the *Decreasing Arousal* profile is possibly reflective of their loss of interest and a concurrent decrease in effortful engagement with the task. This would be consistent with findings on the link between a fixed mindset and lower mental effort^34^ and duration of engagement^35^ during a task. Furthermore, Lou and colleagues^54^ found university students with a fixed mindset profile to be less engaged with coursework than were students characterized by growth and mixed mindset profiles. This interpretation would also be in line with the findings of Tapola and colleagues^3^, who demonstrated that success-oriented fourth to six graders’ self-reported situational interest decreased toward the end of the task, while mastery-oriented students’ interest gradually increased during the task. Alternatively, it is possible that the initially high and subsequently lower arousal displayed by the students in the *Decreasing Arousal* profile reflects initially high and then gradually decreasing state anxiety. Namely, also greater state anxiety is reflected in higher physiological arousal^15^. Goals to avoid demonstrating a lack of ability, which are characteristic of a fixed mindset meaning system, have been associated with higher state anxiety during a stressful task^56^. Additionally, Spangler and colleagues^57^ showed that students reported their state anxiety to be the highest just before an exam and to decrease afterwards.

The gradual increase in arousal in the *Increasing Arousal* profile possibly reflects an increase in the intensity of constructs related to approach motivation (e.g., interest) or effort expenditure during the task^21^. Namely, Tapola and colleagues^3^ found that the self-reported situational interest of mastery-oriented students—characterized by high mastery and lower performance goals—gradually increased during a task. Although, after adjusting for multiple comparisons, the greater than expected number of *Growth Mindset Tendency* students in the *Increasing Arousal* profile did not reach statistical significance, it is possible that both the *Growth* and *Fixed Mindset Tendency* students in this physiological profile became increasingly more interested and effortfully engaged during the task. Interestingly, though, the otherwise gradually increasing arousal in this physiological profile shows a slight decrease in the end of the task. It is possible that, although otherwise becoming increasingly interested and effortfully engaged with the task, after a longer period of time working on the task, the students in this profile started to lose interest in the task due to its repetitive nature. If this decrease in arousal in the end of the task reflects loss of interest, on-task physiological arousal could provide information on optimal lengths of certain learning activities in terms of students’ situational as well as continued interest. Namely, students’ greater situational interest in the end of a task has been shown to predict their interest to do similar tasks in the future^26^.

As to the *Decreasing and Increasing Arousal* profile, the initially high arousal could possibly reflect these students’ concurrent high state anxiety. By the second half of the first block, their arousal had nevertheless already decreased markedly, possibly reflecting a decrease in anxiety^57^, after which their arousal showed a gradual increase to levels similar to those at the beginning of the task. Thus, it is possible that students in the *Decreasing and Increasing Arousal* profile became increasingly engaged with the task once they were able to alleviate their high state anxiety. Nonetheless, as we did not include any self-reported measures, these interpretations remain highly speculative. Still, the results indicate that combining physiological measures with self-reports of state anxiety and constructs related to approach motivation could possibly contribute to a better understanding of temporal patterns of state anxiety.

As expected, physiological profile membership was associated with performance on the task (with this effect being large; H3). Students in the *Increasing Arousal* profile group performed better on the task than students in the *Decreasing Arousal* profile group. As to the version of the task and math grade, there were no differences between the groups (H3). Becoming gradually more interested in a mathematical problem-solving task has been shown to predict students’ performance on the task^25^. Additionally, in the case of better performance, higher on-task SCL has been suggested to reflect effortful engagement with a task^8,17,19^, and greater self-reported mental effort has been shown to associate with higher physiological arousal^21^. As such, this result aligns with the speculation of the increasing SCL in the *Increasing Arousal* profile to be reflective of increases in approach-motivation-related constructs such as interest and effortful engagement. Nevertheless, it is possible that the better performance of students in the *Increasing Arousal* group than in the *Decreasing Arousal* group did not result only from greater effort but also from better arithmetic skills, even though we controlled for previous math grade. While math grades reflect students’ math skills, such grades are a subjective assessment produced by the teacher and are less accurate assessments of specific skills than standardized tests^37^. Therefore, it is plausible that the better performance of students in the *Increasing Arousal* profile is at least partly due to their superior arithmetic skills and not only a result of increased engagement.

Students in the *Decreasing and Increasing Arousal* group, although differing from other groups in patterns of arousal, did not differ in task performance. There were a greater than expected number of *Fixed Mindset Tendency* students in the *Decreasing Arousal* profile, while the *Decreasing and Increasing Arousal* profile was not associated with mindset. This may therefore indicate that students who possess similar skill levels in a domain can differ somewhat in their on-task temporal patterns of arousal because of their general motivational tendencies (here, mindsets). It is possible that students endorsing a fixed mindset do not see the value of sustaining their effort during a task and therefore become gradually less and less engaged with the task, which is possibly reflected in a decrease in their physiological arousal. This refers to the potential of physiological measures in contributing to a better understanding of the dynamics of students’ active engagement with the learning material even when such changes in engagement are not expressed in their visually observable behavior. Namely, all of the participants of the present study completed the arithmetic task according to the instructions, without any notable variation in their visually observable behavior during task completion. Nonetheless, physiological measures indicate temporal changes in the participants’ energetic states during the task as well as individual variability in such changes.

Math grade was not significantly associated with physiological profile membership. As math grade is a subjective assessment made by the teacher, it could be assumed to reflect student’s interest in math^37^, and general interest has been shown to predict temporal changes in on-task interest^27^. Nonetheless, math grades are based on students’ achievement of math learning goals rather than on their interest.

When interpreting the results of the present study, it is important to consider its limitations. First, while we explored fluctuations of physiological arousal during a task, we lacked self-report measures to disentangle aspects of approach and avoidance motivation reflected in such arousal. For example, the students in the *Decreasing Arousal* profile might have experienced high anxiety at the beginning of the task or may simply have lost interest or both. This is a major limitation and future research should seek to disentangle these aspects. Furthermore, using other physiological markers that enable appraisals of threat and challenge to be distinguished, such as cardiovascular responses, could provide more insight^58^. Additionally, we lacked a baseline measure of SCL, which would have allowed us to examine overall task-related increases in arousal compared to that baseline and to study the relationship between on-task fluctuations in arousal and such overall task-related increases^17^. Moreover, we lacked a standardized measure of math skills, which would have allowed us to examine whether a gradual increase in arousal, possibly reflecting increased effortful engagement, was associated with better performance on the task over and above students’ arithmetic skills.

As the calculations in the arithmetic task used in the present study were presented in random order to each participant, the fact that the students were not performing the exact same calculations at the same during the task could also be considered a limitation. Nonetheless, this randomized order enabled us to randomize the within-individual fluctuations of on-task perceived difficulty. Therefore, the average arousal fluctuations observed in the data are reflective of overall temporal trends of arousal when the difficulty of the task is held constant. The present study shows that groups of students with clearly differing patterns of arousal can be identified, although these results should be considered preliminary due to the small sample size. It is possible that we were unable to identify other smaller groups. Therefore, there might be students with patterns of arousal different from those identified here. In the future, larger samples should be considered. The problems resulting from the small sample size also concern the classification of students based on their mindset. Again, larger samples should be considered in future studies to explore whether smaller subgroups of students of this age with differing mindset meaning systems emerge, even when assessing goal endorsement in a tradeoff situation.

Additionally, the specificity of the experimental task prevents us from drawing inferences regarding other tasks and contexts. Future studies could explore the stability of physiological profiles regarding different math tasks, tasks in other subject domains, and the stability of physiological profiles over time. Additionally, future research could explore whether individual differences in physiological arousal during similar tasks are already present during the first years of elementary school or whether such differences only begin to emerge during these years. Furthermore, studies have yet to explore whether such individual differences in temporal patterns of physiological arousal longitudinally predict differences in the trajectories of students’ achievement and general motivational tendencies.

As to practical implications, the present study is limited, but it highlights the importance of considering and further examining individual differences in the dynamics of students’ on-task emotions, motivation-related constructs, and physiological arousal. It is possible that different groups of students could benefit from different support from the teacher during different phases of tasks or activities. For example, some groups of students could benefit more from support at the beginning of the task to help manage their initial anxiety, while others could benefit from support in the middle of the task to help them better sustain their effortful engagement if the task requires persistent practice.

All in all, the present findings contribute to the literature on the dynamics of students’ on-task motivational processes. While previous studies have predominantly relied on self-reports, which are subject to social desirability, are often used retrospectively, or require interrupting participants’ during the activity, this study used a physiological measure that does not rely on the participants’ willingness or ability to report their subjective experience accurately. Furthermore, the data was recorded continuously throughout the entire task without interrupting the participants during the task. As such, the findings highlight the potential of physiological measures to shed light on both the covert as well as dynamic aspects of individuals’ motivational processes as they unfold during specific activities^6,7^. In summary, the study underlines the importance of considering the dynamic aspects of students’ motivational processes as well as the heterogeneity of such dynamics.

### Supplementary Information

Supplementary Information.

## Data Availability

The data supporting the conclusions of this article will be made available by the authors, without undue reservation.
