# Supplementary material for: Changes in physiological arousal during an arithmetic task: profiles of elementary school students and their associations with mindset, task performance and math grade
Source: Sci Rep. 2024 Jan 18;14:1606. doi: 10.1038/s41598-024-51683-7 (PMC10796377; doi:10.1038/s41598-024-51683-7)

Changes in physiological arousal during an arithmetic task: Profiles of elementary school students and their associations with mindset, task performance and math grade

Puusepp, Tammi, Linnavalli, Huotilainen, Laine, Kuusisto & Tirri

Supplementary Figure S1

The challenging math task, indication of perceived difficulty and goal endorsement in a tradeoff situation (each presented on a separate page of the online survey, translated from Finnish).

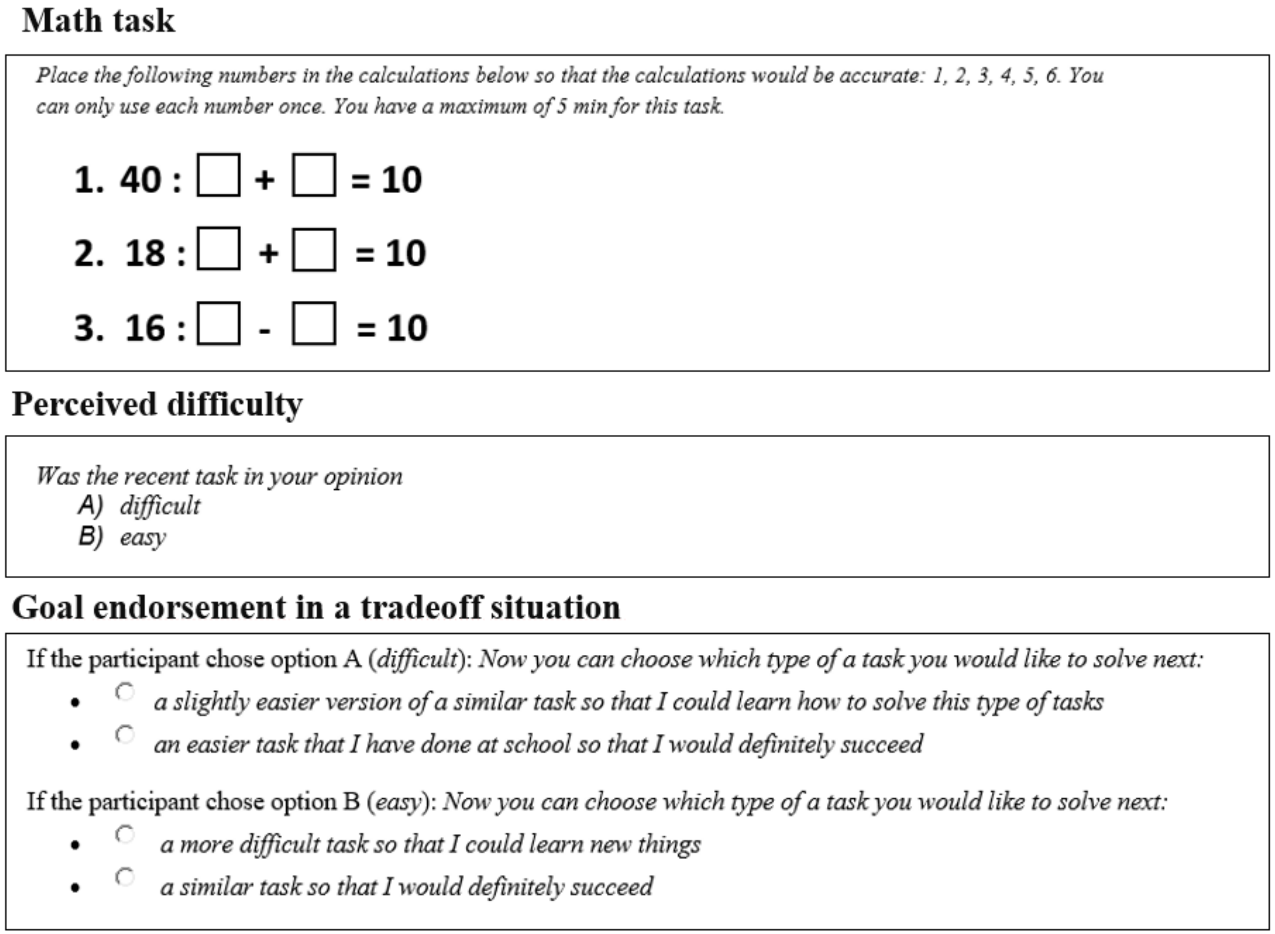

Math task

Place the following numbers in the calculations below so that the calculations would be accurate: 1, 2, 3, 4, 5, 6. You can only use each number once. You have a maximum of 5 min for this task.

1. 40 : ☐ + ☐ = 10
2. 18 : ☐ + ☐ = 10
3. 16 : ☐ - ☐ = 10

Perceived difficulty

Was the recent task in your opinion

A) *difficult*
B) *easy*

Goal endorsement in a tradeoff situation

If the participant chose option A (*difficult*): *Now you can choose which type of a task you would like to solve next:*

- ○ *a slightly easier version of a similar task so that I could learn how to solve this type of tasks*
- ○ *an easier task that I have done at school so that I would definitely succeed*

If the participant chose option B (*easy*): *Now you can choose which type of a task you would like to solve next:*

- ○ *a more difficult task so that I could learn new things*
- ○ *a similar task so that I would definitely succeed*

Supplementary Table S1

Fit indices of the LPA solutions of physiological arousal based on participants with complete SCL data

Number of profiles	Log likelihood	AIC	BIC	SABIC	BLRT *p*-value	Entropy
1	−329.95	675.91	693.66	668.47	-	1.00
2	−252.39	530.78	559.63	518.70	.01	.98
3	−233.05	502.11	542.06	485.38	.01	.95
4	−219.08	484.17	535.22	462.79	.01	.95
5	−203.79	463.58	525.73	437.55	.01	.93

Supplementary Figure S2

Line graphs comparing physiological profiles regarding SCL from solutions with one to five profiles (based on participants with complete SCL data)

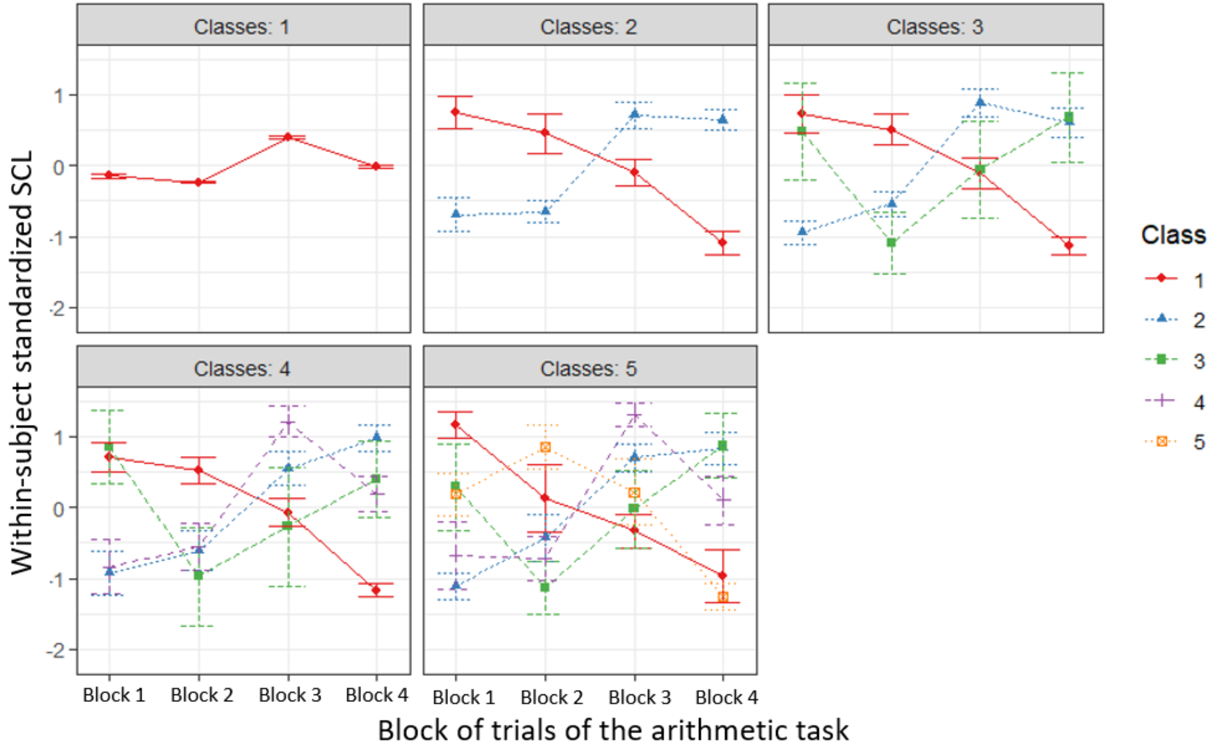

Supplementary Figure S3

Bar plot representing the distribution of gender across physiological profiles

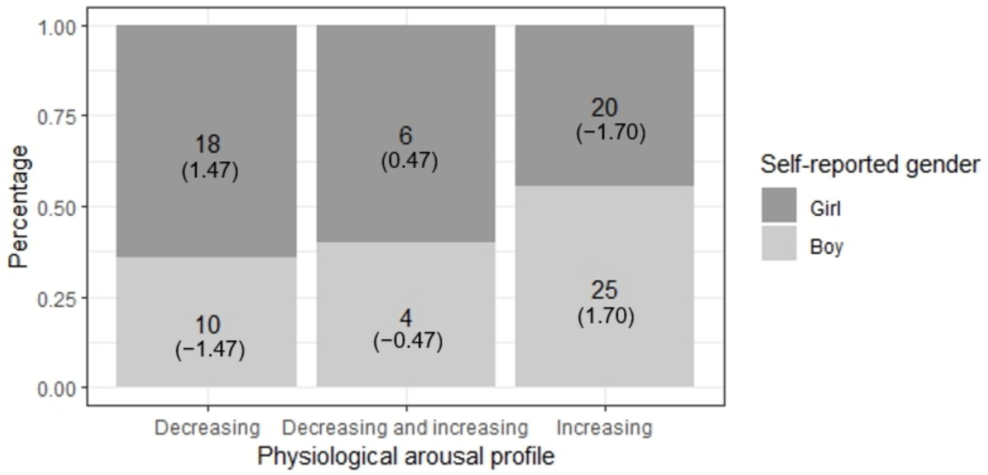

Note. The figure displays observed counts with adjusted standardized residuals indicated in brackets. Crosstab with Fisher's exact test indicated no significant association between gender and physiological profile membership (p = .252; φ_c = 0.19). The adjusted standardized residual cutoff indicating statistical significance adjusted to ±2.64 based on the number of cells.

Supplementary Figure S4

Bar plot representing the distribution of the school of attendance across physiological profiles

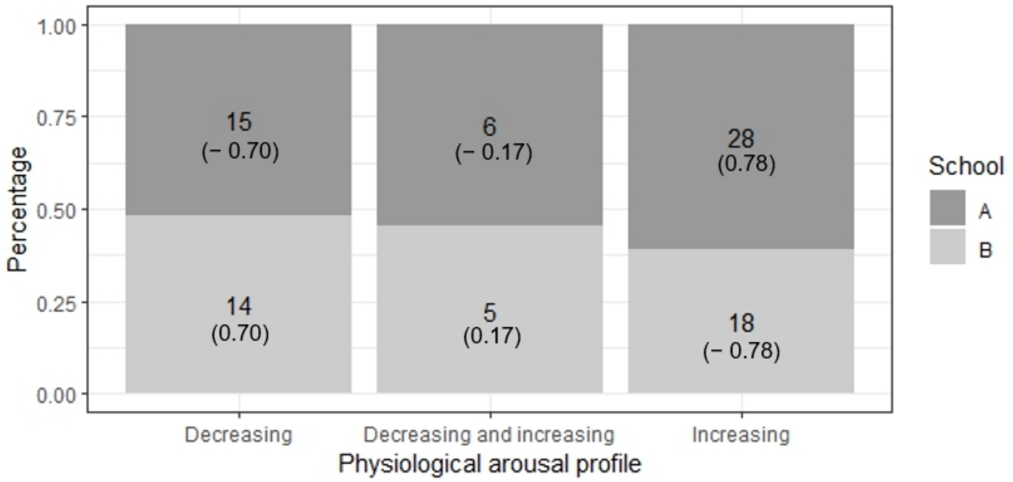

Note. The figure displays observed counts with adjusted standardized residuals indicated in brackets. Crosstab with Fisher's exact test indicated no significant association between school of attendance and physiological profile membership (p = .749; φ_c = 0.09). The adjusted standardized residual cutoff indicating statistical significance adjusted to ±2.64 based on the number of cells.

Supplementary Figure S5

Distributions of the within-subject standardized SCL averages of the three physiological profiles during the four trial blocks

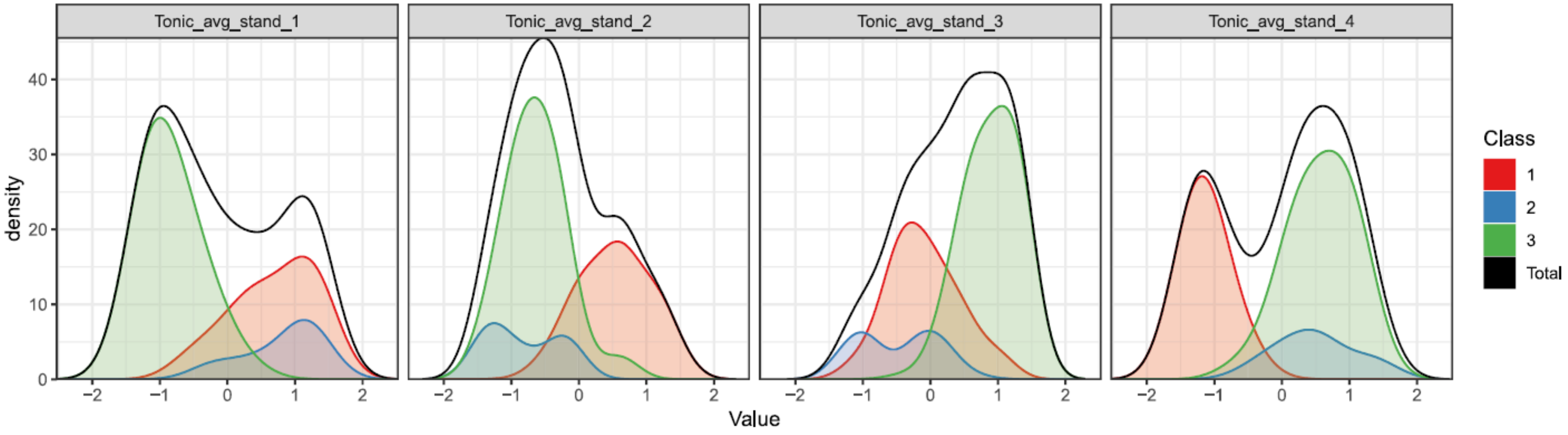

Note. Class 1 – *Decreasing Arousal*, Class 2 – *Decreasing and Increasing Arousal*, Class 3 – *Increasing Arousal*.

Supplementary Figure S6

Posterior probabilities of mindset profile membership

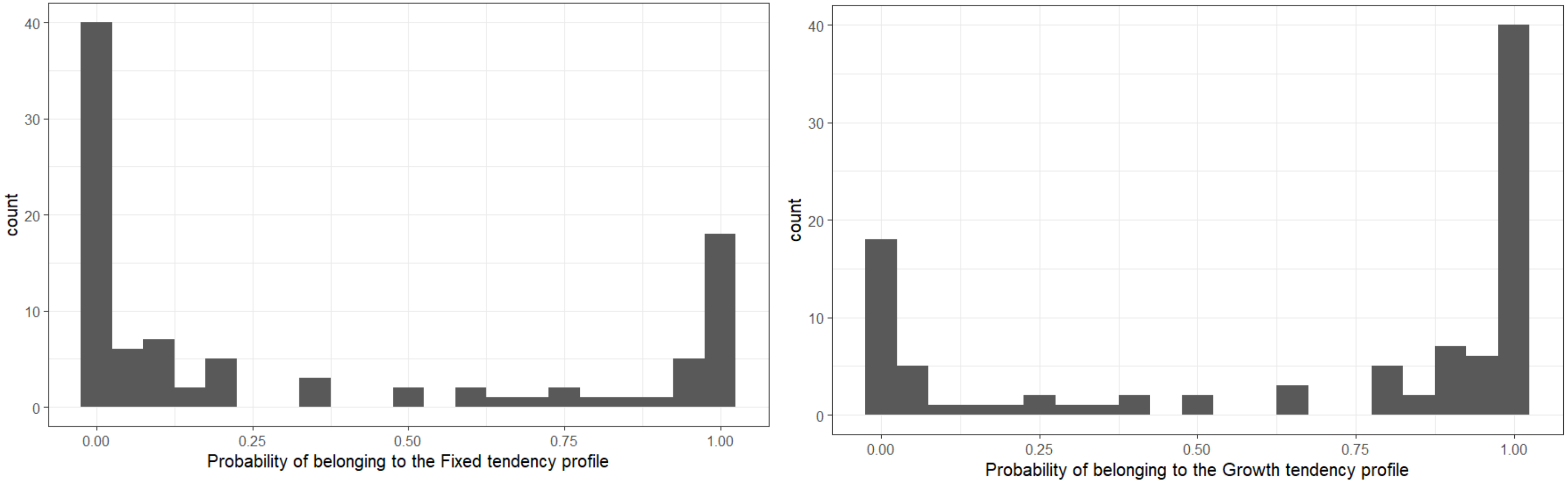

Supplementary Figure S7

Distributions of the mindset profile indicators

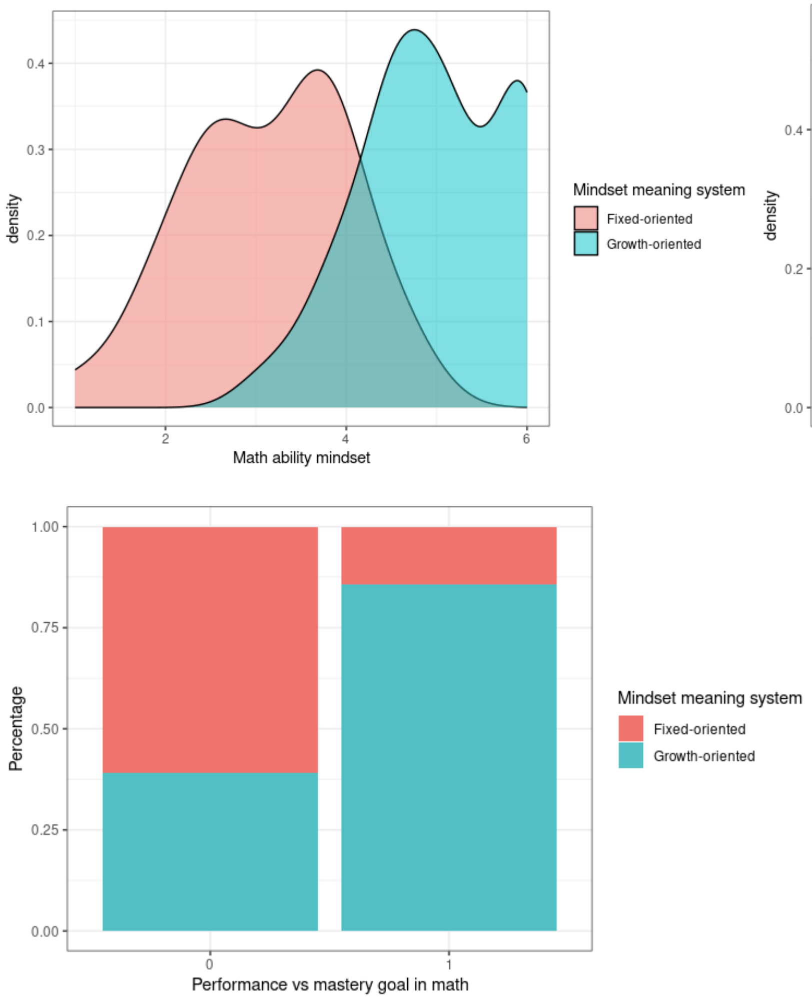

Supplement: Supplementary file 1 — Supplementary Information. [file 41598_2024_51683_MOESM1_ESM.pdf]
